# Leukocytoclastic Vasculitis as a Rare Dermatologic Extraintestinal Manifestation of Ulcerative Colitis in an Elderly Patient with Positive PR3-ANCA

**DOI:** 10.1155/2021/5767699

**Published:** 2021-09-23

**Authors:** Jacky Ng, David Zezoff, Hanadi Abou Dargham

**Affiliations:** ^1^Touro University California, 1310 Club Dr, Vallejo, CA 94592, USA; ^2^Graduate Medical Education, St. Joseph's Medical Center, 1800 N California St, Stockton, CA 95204, USA

## Abstract

Ulcerative colitis is an inflammatory bowel disease that in rare cases may develop extraintestinal manifestations. This case report aims to add to the limited clinical data on leukocytoclastic vasculitis and possible ANCA-associated vasculitis as rare cutaneous and rheumatologic extraintestinal manifestations of IBD, particularly in elderly patients. Our case involves a 79-year-old male with a history of mild-moderate ulcerative colitis on oral mesalamine 2.4 g daily and pyoderma gangrenosum who presented with recurrent bilateral polyarthralgia, joint swelling, diffuse lower extremity purpura, acute kidney injury, and scrotal rash. Autoimmune titers were significant for positive ANA and PR3-ANCA. Biopsy of purpuric lesions demonstrated findings suggestive of leukocytoclastic vasculitis. The patient was promptly treated with pulse-dose methylprednisolone for 3 days with rapid improvement of symptoms.

## 1. Introduction

Ulcerative colitis (UC) is an inflammatory bowel disease (IBD) characterized by autoimmune damage to the colonic mucosa. It most commonly involves the rectum and may extend proximally to other portions of the colon. UC is known to have unique extraintestinal manifestations that frequently affect the joints, skin, or eyes. [1] However, it is rare for the coexistence of small-vessel vasculitis and these enteropathic symptoms without active bowel disease. [2] In our case, we present a 79-year-old male with a significant history of mild-moderate UC and pyoderma gangrenosum who presented with diffuse polyarthralgia with swelling, extensive bilateral lower extremity purpura, scrotal ulcerations, and acute kidney injury. Serological testing was significant for PR3-ANCA antibodies, and dermatological evaluation of the purpura was consistent with leukocytoclastic vasculitis. The patient was treated with pulse-dose methylprednisolone for 3 days with rapid improvements in arthralgias, bilateral leg rash, and renal function. The patient was eventually discharged to a skilled nursing facility for rehabilitation of deconditioning and advanced medical needs.

## 2. Case Description

**Figure 1:**
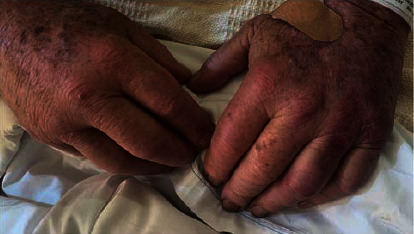
The patient had been prescribed oral mesalamine 2.4 g twice a day in the past as a treatment for his ulcerative colitis, but the patient admitted to noncompliance due to inability to care for himself.

**Figure 2:**
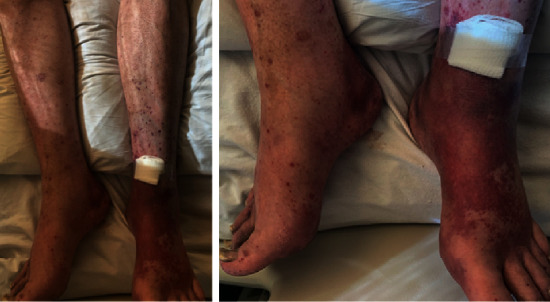
Status post skin biopsy, initiation of pulse-dose Solu-Medrol.

A 79-year-old male with a significant history of mild-moderate ulcerative colitis on oral mesalamine 2.4 g daily and pyoderma gangrenosum, hypertension, coronary artery disease, and benign prostatic hypertrophy presented to the emergency department for evaluation of bilateral polyarthralgia with associated weakness and swelling (including the metacarpal joints and ankles; see Figure 1), diffuse lower extremity purpura, violaceous rash (see Figure 2), and scrotal ulcerations. He had similar pains 3 months prior to presentation, but symptoms resolved without medical intervention. Notably, he reported the rash on his lower extremities started one week prior to presentation and was initially pruritic. However, this too gradually abated.

Initial lab work demonstrated acute kidney injury with a creatinine of 3.6 mg/dL (normal range: 0.7–1.3 mg/dL). Urinalysis was positive for gross hematuria and proteinuria. Coagulation studies showed elevated fibrinogen levels at 882 mg/dL (normal range: 200–393 md/dL), elevated PT/INR at 16.2 and 1.4, respectively, and elevated PTT at 41.4. Inflammatory markers such as ESR and CRP were also elevated at 84 mm/hr (NR < 20 mm/hr) and 32.53 mg/dL (NR: 0.01–0.82 mg/dL), respectively. CT abdomen and pelvis without IV contrast demonstrated no evidence of diverticulitis, colitis, or ischemic bowel disease, no mechanical bowel obstruction, perforation, or free air, + air-fluid levels within the rectum without wall thickening, marked distention of the urinary bladder, and prostatomegaly. CT chest w/o contrast and CXR were unremarkable.

**Figure 3:**
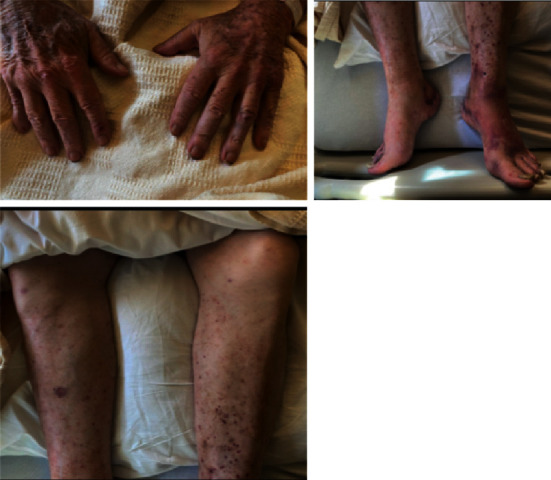
3 days post-Solu-Medrol treatment with improvement in purpura and arthralgias.

Given these diagnostic findings and the patient's subacute onset purpura with confluence below the waist and worsening bilateral MCP, shoulder, knee, and dorsal foot joint swelling and pain, rheumatology was consulted. Autoimmune/vasculitis-related titers were sent, which were grossly unremarkable except for positive antinuclear antibodies (ANA), anti-chromatin/nucleosome antibodies, and proteinase-3 antineutrophil cytoplasmic antibodies (PR3-ANCA/c-ANCA). Nephrology was onboard due to concerns for pauci-immune glomerulonephritis, but the patient did not exhibit symptoms specific to granulomatosis polyangiitis (GPA), such as nasal or respiratory complications. Additionally, no casts were present in urinalysis. Because of the patient's age and elevated PSA, he was not a candidate for cyclophosphamide or any other immunosuppressive therapy. Rheumatology postulated the cause to be extraintestinal manifestations of poorly controlled ulcerative colitis, but due to the unclear diagnosis, skin biopsy was taken by general surgery and sent to pathology. The patient was treated empirically with high-dose Solu-Medrol for 3 days. Patient's joint pain, skin rash (see Figure 3), and kidney function continued to improve day to day under the high-dose Solu-Medrol regimen. Final pathology of left leg skin biopsy, along with clinical correlates, demonstrated findings compatible with leukocytoclastic vasculitis. The patient was discharged to SNF for rehabilitation, and it was recommended that he follows with his primary care, gastroenterologist, cardiologist, and rheumatologist. Unfortunately, the patient shortly thereafter was infected with the deadly virus SARS-CoV-2 and was placed on hospice after he developed complications of COVID-19.

## 3. Discussion

Ulcerative colitis (UC) is an inflammatory bowel disease (IBD) that commonly causes continuous colonic inflammation and lesions, but is also known to be associated with extraintestinal manifestations (EIM) including rash, such as pyoderma gangrenosum or erythema nodosum, arthritis, primary sclerosis cholangitis, ulcerations, and more. [3] The pathogenesis of EIM in IBD is still unclear, but is believed to be involved with an upregulated immune response at the extraintestinal site due to shared epitopes [1]. In the literature, multiple EIM may occur simultaneously and the presence of one EIM can confer a higher likelihood to develop further additional EIM [1]. It is reported that skin manifestations occur in about 15% of patients with IBD and is most commonly associated with pyoderma gangrenosum and erythema nodosum [4, 5]. Joint symptoms affecting peripheral large and small joints occur in up to 40% of patients with IBD and typically demonstrate minimal to no erosions [6–8]. They are classified into 2 types: type 1 (pauciarticular) arthralgia, which is typically asymmetric/migratory involvement affecting less than 5 large joints, or type 2 (polyarticular) arthralgia, which is frequently symmetrical involving 5 or more small joints. Type 1 is associated with active intestinal disease activity and high frequency of other EIM, whereas type 2 may be independent of active intestinal symptoms and is associated only with uveitis [6, 8]. Additionally, type 1 is usually self-resolving or improves with anti-inflammatory treatment whereas type 2 symptoms may be refractory to anti-inflammatory treatment and can persist for months to years. In our case, based on the above evidence and the patient's previous history of pyoderma gangrenosum, it is found that our patient presented with type 1 pauciarticular arthralgia.

A unique manifestation of our patient's ulcerative colitis flare-up was the vasculitis involvement with leukocytoclastic vasculitis (LCV). Leukocytoclastic vasculitis manifests as an exceedingly rare palpable, purpuric skin lesion, typically of the lower extremities, which was representative in our case [9, 10]. As of 2014, less than 20 cases of LCV have been reported in relation to UC in the English literature [11]. Upon the review of these cases in the literature, it was noticed that LCV involved juvenile to young adult patients; on the other hand, LCV manifested in an elderly patient in our case [11–14]. It appears that there is no apparent pattern in whether LCV precedes or succeeds intestinal symptoms [15]. Blanco et al. reported LCV as an immune complex-mediated disease in which the deposition of immune complexes within the subendothelial space in the wall of small dermal vessels leads to a cascade of events, triggering the activation of complement, leukostasis, release of lysosomal enzymes, and consequent rupture and extravasation of erythrocytes and tissue necrosis [16]. Ulcerative colitis shares a similar pathogenesis in which immune mechanisms and deposition of immune complexes occur in the intestinal mucosa instead of the vascular wall [17]. This would support the theory of how treating the underlying UC results in LCV remittance. However, the exact relationship between LCV and UC is still in debate [1, 13]. Miossec et al. proposed such a relational mechanism, stating that unregulated Th17 responses from interleukin-21 enhanced cytolytic activity as the culprit linking UC and LCV [18]. Subsequent downstream effects contribute to inflammation, ischemia, and impaired mucosal healing, resulting in intestinal and dermal damage [19].

Ulcerative colitis is typically associated with pANCA, which are antibodies directed against myeloperoxidase (MPO-ANCA), and is specific for microscopic polyangiitis. However, in this case, cANCA, or antibodies against proteinase-3 (PR3-ANCA), was demonstrated. cANCA is commonly associated with granulomatosis with polyangiitis (formerly Wegener's granulomatosis), for which this diagnosis was plausible [20]. Recent literature has used cANCA as a biomarker differentiating UC from CD [21, 22], and it has been associated with various systemic diseases such as lupus erythematosus (SLE), rheumatoid arthritis, IBDs, endocarditis, and other infections and malignancies [23]. To our knowledge, the current literature detailing the specific role of cANCA in relation to positive ANA and anticentromere antibodies is limited. However, cANCA positivity in this case likely represents UC with extensive disease as supported by Mahler et al [24]. This would be in concordance with the patient's lack of medical adherence with mesalamine coupled with his multiple EIMs. The argument supporting the differential of a pauci-immune glomerulonephritis, such as granulomatosis with polyangiitis, is less likely. The classical presentation of GPA typically involves nasal, pulmonary, or confirmed renal involvement, which were absent in this case [25]. The patient's acute kidney injury rapidly improved with resolution of his urinary retention after Foley catheter insertion, ruling out a glomerular cause of injury. A kidney biopsy was not performed during his hospital stay given the rapid improvement in his kidney function post steroid therapy.

## 4. Conclusion

Our case report adds to the limited clinical data on leukocytoclastic vasculitis and possible ANCA-associated vasculitis as rare cutaneous and rheumatologic extraintestinal manifestations of IBD, particularly in elderly patients. Patients with a history of ulcerative colitis presenting with a clinical picture of purpuric skin lesions, polyarthralgias, and acute kidney injury should be promptly evaluated for rare extraintestinal manifestations of inflammatory bowel disease. Definitive treatment of patient's underlying UC with high-dose systemic corticosteroids led to marked improvement of LCV, arthralgias, and kidney function.
